# Association between periodontitis and oral health–related quality of life in patients with systemic conditions: A meta‐analysis

**DOI:** 10.1002/jper.70130

**Published:** 2026-04-18

**Authors:** Rafael Vargas Bortolaso, Catiusse Crestani Del' Agnese, Leandro Machado Oliveira, Patricia A. Miguez, Fabricio Batistin Zanatta, Raquel Pippi Antoniazzi

**Affiliations:** ^1^ Postgraduate Program in Dentistry, Department of Stomatology, Emphasis on Periodontics Federal University of Santa Maria Santa Maria Brazil; ^2^ Department of Periodontology, Endodontics and Dental Hygiene, Adams School of Dentistry The University of North Carolina at Chapel Hill Chapel Hill North Carolina USA

**Keywords:** comorbidity, periodontitis, quality of life, systematic review

## Abstract

**Background:**

The objective of this systematic review is to evaluate whether periodontitis is associated with impaired oral health–related quality of life (OHRQoL) in adults with systemic conditions.

**Methods:**

Five databases were searched to identify observational studies that tested the association between periodontitis (diagnosed by clinical periodontal examination) and OHRQoL (validated instrument). Random‐effects models were applied in the meta‐analyses, and the Newcastle‐Ottawa Scale (NOS) was used for quality assessment.

**Results:**

Seventeen studies were included, with 94.1% using the OHIP‐14 instrument. Only one study was rated as high quality. Meta‐analysis showed a small and significant association between periodontitis and impaired OHRQoL (SMD: 0.31; 95% CI: 0.03–0.59). Individuals with periodontitis had higher OHIP‐14 scores than the comparison group (MD: 2.29; 95% CI: 0.24–4.33). Studies using the 2017 periodontitis classification yielded significantly higher estimates than those using earlier criteria. Furthermore, a strong negative impact of periodontitis on OHRQoL was observed among individuals with psoriasis, whereas no significant association was identified for other systemic conditions.

**Conclusions:**

Periodontitis has a small negative impact on OHRQoL in patients with comorbidities. The type of systemic condition and the periodontitis diagnostic criteria influence this association.

**Plain language summary:**

Periodontitis negatively impacts oral health–related quality of life across different populations. However, this impact may appear smaller in individuals with certain systemic conditions, as additional health challenges can overshadow the perceived effects of periodontitis on daily life. Our systematic review found a small negative impairment of OHRQoL associated with periodontitis in patients with comorbidities, with the magnitude of this association influenced by the type of systemic condition and the diagnostic criteria used to define periodontitis. Further high‐quality studies are needed to strengthen the current evidence.

## 1 INTRODUCTION

Quality of life was defined by the World Health Organization as “individuals' perception of their position in life in the context of the culture and value systems in which they live and in relation to their goals, expectations, standards and concerns”.^1^ Likewise, an oral health–related quality of life (OHRQoL) is a complementary measure to oral clinical diagnosis, which determines a multidimensional perception of biological, social, psychological, and cultural characteristics and provides a subjective assessment of the individual's view of their oral health.^2^, ^3^

Periodontitis is characterized as a prevalent chronic noncommunicable disease and as being the main cause of tooth loss in adults.^4^ The severe form is a significant contributor to the global burden of oral disease and affects 12.5% of the global adult population.^5^ The pathogenesis of periodontitis is determined by the interaction between the dysbiotic dental biofilm and the immune‐inflammatory response of the susceptible host and may be modified by environmental, genetic, and/or acquired conditions such as smoking and systemic diseases.^6^ Periodontitis can progress to loss of tooth support tissues, which can determine tooth mobility and loss, impaired mastication, pain, and functional, esthetic, and social problems.^7^, ^8^ These consequences are related to a negative impact on the perception of OHRQoL.^9^

Some systemic diseases can negatively impact periodontal health^6^, ^10^ and consequently OHRQoL.^11^, ^12^, ^13^, ^14^ In these populations, OHRQoL can be affected by oral diseases and the aggravations of systemic diseases.^8^, ^9^, ^15^ On the other hand, although periodontitis is more prevalent in individuals with some comorbidities, it may determine a smaller impact on OHRQoL, due to the important vulnerability of this population, which has a greater overall disease burden, with greater severity and more debilitating outcomes than those related to periodontitis. Some studies investigating systemic diseases and conditions such as leukemia, ankylosing spondylitis, rheumatoid arthritis, or organ transplantation have not shown an association between periodontitis and impaired OHRQoL.^16^, ^17^, ^18^, ^19^, ^20^, ^21^ A recent systematic review showed that the magnitude of the association between periodontitis and OHRQoL seems to be reduced in individuals with comorbidities.^22^

To our knowledge, no systematic review has evaluated the association between periodontitis and OHRQoL, specifically considering individuals with various comorbidities. Determining this association would allow a better understanding of patients' perceptions during the course of systemic conditions, assisting in the appropriate management of education and care in oral health. Therefore, this systematic review aimed to assess the association between periodontitis and OHRQoL in adults with systemic conditions and to investigate relevant factors that may influence this association.

## 2 MATERIALS AND METHODS

This systematic review was conducted in accordance with the PRISMA guidelines^23^ (see Table S1 in online *Journal of Periodontology*) and the review protocol was registered in the PROSPERO (number CRD42024522723).

### 2.1 Focused question and inclusion criteria

The focused research question was: “What is the association between periodontitis and OHRQoL in patients with systemic conditions?”. The PECOS strategy consisted of the following acronym: Participants (P): adult (18 years or older) with systemic conditions or diseases; Exposure (E): periodontitis or severe periodontitis (as defined by the authors) diagnosed using periodontal probing depth (PPD) and/or clinical attachment level (CAL); Comparison (C): absence of periodontitis or milder forms of periodontitis; Outcomes (O): OHRQoL measured using validated instruments^24^; Study (S): Observational studies (longitudinal, case‐control, or cross‐sectional).

### 2.2 Exclusion criteria

Studies with exclusive assessment of children and adolescents, without distinction of data between participants with systemic diseases and healthy, with periodontitis diagnosed by self‐reported or through radiographic parameters and unclear definition of periodontitis were excluded. Furthermore, literature reviews, intervention studies, or the studies lack data for continuous meta‐analysis (measures of central tendency and dispersion) or lack effect measures for binary meta‐analysis were also excluded.

### 2.3 Information sources search

Two reviewers (C.C.D. and R.P.A.) independently and in duplicate conducted an electronic literature search as well as a hand search to identify eligible studies. Disagreements between the reviewers were resolved by discussion. If a disagreement persisted, the judgment of a third reviewer (F.B.Z.) was considered decisive. Searches were performed in the PubMed, EMBASE, LILACS, Web of Science, and Scopus databases for relevant studies published, without language restrictions or year of publication up to January 2025. Grey literature was searched through the DANS database. Additionally, the same two reviewers manually searched the reference lists of relevant articles and systematic reviews. The search strategies for all electronic databases and grey literature are detailed in Table S2 in the online *Journal of Periodontology*. The selected studies were compared to avoid duplicate data.

The two reviewers (C.C.D. and R.P.A.) received training to ensure consistency in applying the inclusion and exclusion criteria. In the first phase, they independently assessed titles and abstracts using the criteria. In the second phase, the same two reviewers independently applied the criteria based on full‐text readings. Inter‐examiner agreement during the full‐text analysis phase was nearly perfect (Kappa coefficient = 0.96).

### 2.4 Data extraction

Data from the included studies were extracted in duplicate by two reviewers (C.C.D. and R.P.A.). The following data were extracted from the included studies: authors, year of publication, country, study design, sample size, age, sex, systemic conditions, definition of periodontitis, comparison group, OHRQoL assessment instrument, adjustment variables in multivariate analysis, and OHRQoL domains impacted by periodontitis.

### 2.5 Quality assessment

The quality assessment of included studies was conducted in duplicate by two reviewers (C.C.D. and R.P.A.) using the Newcastle‐Ottawa Scale (NOS)^25^, adapted for cross‐sectional studies,^26^, ^27^ in accordance with the purpose of this systematic review (see Table S3 in online *Journal of Periodontology*). One study, originally labeled as case‐control by its authors,^28^ was considered in our review as cross‐sectional with a control group due to selection criteria based on exposure rather than on outcome. The tool consists of seven items and four domains: selection (maximum of two stars); comparability of study groups (maximum of one star); exposure (maximum of two stars); and outcome (maximum of four stars). The score range is from 0 to 9 points, categorized as low (0–3 points), moderate (4–6 points), and high quality (7–9 points).^27^

### 2.6 Grading of certainty of evidence

The Grading of Recommendations Assessment, Development, and Evaluation (GRADE) system was used to rank the evidence by two examiners (C.C.D. and R.P.A.).^29^ For cross‐sectional studies, the certainty of the evidence starts at low and can be upgraded or downgraded. Downgrading was based on limitations in quality, inconsistency, indirectness, imprecision, and publication bias. The presence of a large effect, dose–response gradient, and no plausible confounding promote upgrading. A summary of the results, including the evidence profile (number of studies, evaluated domains, and interpretation) with polled estimates and the overall GRADE score (see Table S4 in online *Journal of Periodontology*).

### 2.7 Data analysis

All analyses were performed using R software (version 4.3.3) and RStudio (version 2023.12.1) along with the *“meta”* and *“metafor”* packages. Continuous data from all OHRQoL assessment instruments and OHIP‐14 (Oral Health Impact Profile) were pooled respectively in standardized mean difference (SMD) and mean difference (MD) meta‐analyses. Dichotomized OHRQoL data, as defined by the authors of each study, which resulted in an effect measure (odds ratios, OR), were pooled in a binary meta‐analysis (converted into a log‐binomial [log] scale). When more than one periodontitis severity category was reported as exposure, only the most severe was included in the meta‐analysis. Effect sizes and respective 95% confidence intervals were estimated using the inverse variance method, DerSimonian‐Laird estimator for Tau^2^, using random‐effects model. Heterogeneity was assessed using the Cochran Q test (*p* < 0.05) and the *I^2^* inconsistency test. Meta‐regression and subgroup analyses were performed to identify possible sources of variability between studies. The percentage of heterogeneity explained by the set of variables was obtained by the adjusted *R^2^* of the meta‐regression model. A funnel plot was constructed to assess publication bias (visually) and Egger's statistical test. Effect magnitude (SMD) in continuous outcome was interpreted as trivial (0 to 0.2), small (0.2 to 0.5), moderate (0.5 to 0.8), and large (> 0.8), and in binary outcome (OR) such as trivial effect (1.01 to 1.68), small (1.68 to 3.47), moderate (3.47 to 6.71), and large (> 6.71).^30^, ^31^

## 3 RESULTS

### 3.1 Search results and study characteristics

**FIGURE 1:**
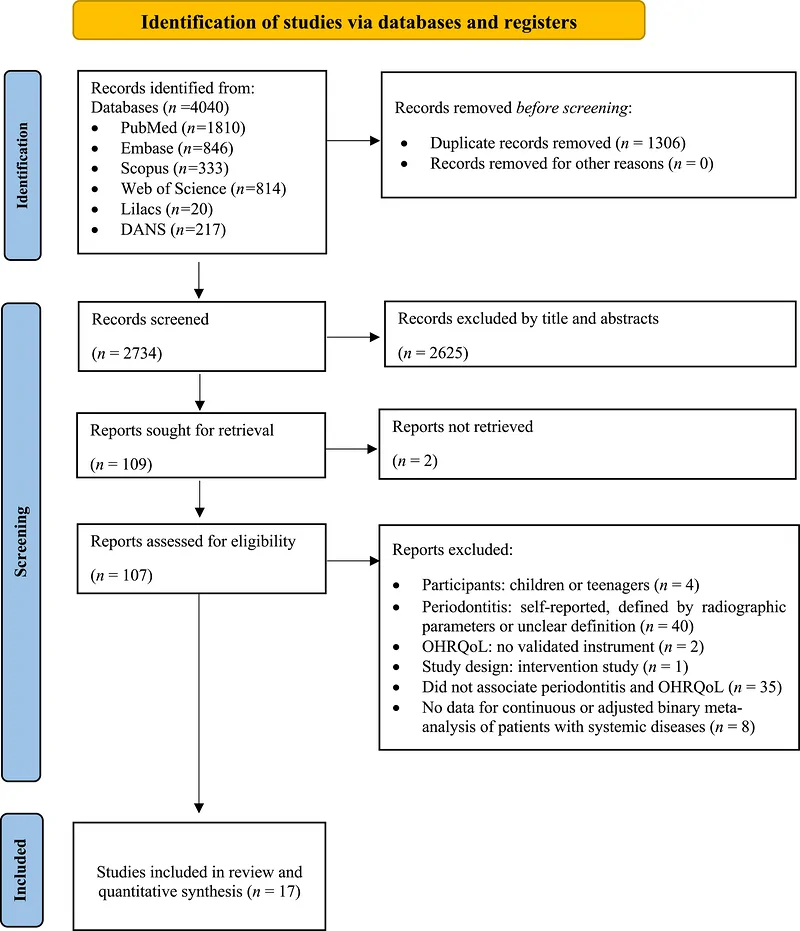
Flowchart for the study selection procedure.

The flowchart summarizing the selection process is depicted in Figure 1. Screening was performed of 4040 titles/abstracts and 107 potentially eligible records were submitted to full‐text analysis. A total of 17 studies were included. Of these studies, 14 reported only continuous OHRQoL outcomes, one reported exclusively binary OHRQoL data,^32^ and two provided both continuous and binary data.^28^, ^33^ No additional studies were identified during the manual search. The studies and reasons for exclusion after full‐text analysis are shown in Table S5 in the online *Journal of Periodontology*. No additional studies were identified through manual search.

The general characteristics of the included studies are shown in Table 1. All studies were cross‐sectional and used the OHIP‐14 instrument, except one case‐control with OIDP.^28^ No longitudinal studies were identified for the research question. Eight studies evaluated the OHRQoL domains in the OHIP‐14. Of these, periodontitis was associated with the domains physical pain (*n* = 4), functional limitation (*n* = 4), physical disability (*n* = 5), psychological discomfort (*n* = 4), psychological disability (*n* = 4), social disability (*n* = 1), and handicap (*n* = 3). In two studies, periodontitis was not associated with any domain.^21^, ^33^ Six studies^16^, ^17^, ^18^, ^19^, ^21^, ^34^ defined the case of periodontitis according to Page and Eke,^35^ four^12^, ^20^, ^36^, ^37^ according to Eke et al.,^38^ and four^13^, ^14^, ^28^, ^33^ used the 2017 EFP/AAP classification.^4^ Three studies reported significant adjusted associations (*p* < 0.05) between periodontitis and OHRQoL treated as a continuous outcome,^12^, ^14^, ^52^ while two studies reported a non‐significant association.^20^, ^36^ Additionally, three studies presented adjusted analyses using OHRQoL as a binary outcome.^28^, ^32^, ^33^ In the quality appraisal, seven studies (41.2%), nine studies (52.9%), and one study (5.9%)^32^ were rated as low, moderate, and high quality, respectively (see Table S6 in online *Journal of Periodontology*).

**TABLE 1:** Main characteristics and OHRQoL domains impacted by periodontitis in the 17 included studies.

Author Country Study design	Total sample size (T) E and C (Mean age; % women)	Systemic condition OHRQoL instrument	Exposure (E): prevalence (Diagnosis of periodontitis) Comparison (C)^*^	Association between periodontitis and OHRQoL adjusted for confounding factors	Domains impacted by periodontitis
Falcón‐Flores et al. 2024 ^37^ Mexico Cross‐sectional	T: 79 (60.4; 77.2%) E: 56 (NR) C: 9 (NR)	Type 2 diabetes mellitus OHIP‐14	E: Severe periodontitis: 70.9% (Eke et al. 2012)^38^ C: No periodontitis	Unadjusted	NR
Mishra et al. 2024 ^13^ India Cross‐sectional	T: 100 (NR) E: 50 (43.7; 26.5%) C: 50 (41.4; 24.5%)	Psoriatic arthritis OHIP‐14	E: Generalized stage II/III/IV periodontitis: 50.0% (2017 EFP/AAP)^4^ C: No periodontitis	Unadjusted	Physical pain, functional limitation, physical disability, psychological discomfort, psychological disability
Posada‐López et al. 2023 ^33^ Colombia Cross‐sectional	T: 52 (54.5; 81.4%) E: 31 (NR) C: 21 (NR)	Rheumatoid arthritis OHIP‐14	E: Periodontitis: 59.6% (2017 EFP/AAP)^4^ C: No periodontitis	Adjusted: Sex, age, socioeconomic status, practicing exercise, type of rheumatoid arthritis, remission, erosive rheumatoid arthritis, swelling, pain, morning stiffness, alternative therapies	No domain affected
Kabisch et al. 2022 ^52^ Germany Cross‐sectional	Type 1 diabetes: T: 80 (48.0;48.4%) E: 48 (49.0; 45.8%) C: 32 (50.0; 59.3%) Type 2 diabetes: T: 381 (67.0; 51.3%) E: 228 (68.0; 43.4%) C:153 (67.0; 54.2%)	Type 1 diabetes mellitus and Type 2 diabetes mellitus OHIP‐14	E: Periodontitis: 60.0% and 59.8% (CPI > 2 in at least one oral sextant) C: No periodontitis	^#^Adjusted: Sex, age, type of diabetes, diabetes onset and duration, neuropathy, nephropathy, retinopathy, HbA1c, medication, insulin treatment, depression, hypertension, body mass index, cholesterol levels, smoking status, poor dental hygiene, infrequent dentist appointments, problem areas in diabetes	NR
Lazureanu et al. 2022 ^14^ Romania Cross‐sectional	T: 147 (NR) E: 114 (NR) C: 33 (NR)	Cardiovascular disease OHIP‐14	E: Periodontitis: 77.6% (2017 EFP/AAP)^4^ C: No periodontitis	^#^Adjusted: Oral hygiene, body mass index	Physical pain, functional limitation, physical disability psychological disability, social disability, handicap
Costa et al. 2021 ^28^ Brazil Case‐control	T: 295 (49.4, 61.4%) E: 121(NR) C: 174 (NR)	Psoriasis OIDP	E: Stage II/III/IV periodontitis: 41.0% (2017 EFP/AAP)^4^ C: No periodontitis	^#^Adjusted: Diabetes, psoriasis, anxiolytics use	NR
Angst et al. 2020 ^36^ Brazil Cross‐sectional	T: 55 (42.1, 27.3%) E: 13 (51.8, 23.1%) C: 24 (31.3, 29.2%)	Leukemia OHIP‐14	E: Severe periodontitis: 23.6% (Eke et al. 2012)^38^ C: No/mild periodontitis	Adjusted: Hospitalization, gingivitis	Psychological discomfort
Han et al. 2020 ^20^ Malaysia Cross‐sectional	T: 87 (NR) E: 29 (55.0, 75.9%) C: 58 (54.7, 89.7%)	Rheumatoid arthritis OHIP‐14	E: Periodontitis: 33.3% (Eke et al. 2012) ^38^ C: No periodontitis	Adjusted: Sex, age, education level, brushing frequency	Functional limitation, physical disability, handicap
Oliveira et al. 2020 ^12^ Brazil Cross‐seccional	T: 98 (52.0, 45.0%) E: 74 (NR) C: 24 (NR)	End‐stage renal disease (Hemodialysis) OHIP‐14	E: Severe periodontitis: 41.1% (Eke et al. 2012) ^38^ C: No periodontitis	^**^Adjusted: Skin color, age, schooling, smoking status, visible plaque index	Physical pain, physical disability, psychological discomfort, psychological disability
Schmalz et al. 2020 ^21^ Germany Cross‐sectional	T: 39 (55.6, 51.3%) E: 12 (NR) C: 6 (NR)	Acute leukemia OHIP‐14	E: Severe periodontitis: 30.8% (Page and Eke, 2007)^35^ C: No/mild periodontitis	Unadjusted	No domain affected
Sousa et al. 2019 ^32^ Brazil Cross‐sectional	T: 302 (63.1, 71.2%) E: 116 (NR) C: 42 (NR)	Diabetes mellitus OHIP‐14	E: Periodontitis: 38.4% (AAP, 1999)^53^ C: No periodontitis	^**^Adjusted: Denture need, xerostomia	Physical pain, functional limitation, physical disability, psychological discomfort, psychological disability, handicap
Schmalz et al. 2018 ^17^ Germany Cross‐sectional	T: 50 (47.2, 48.0%) E: 23 (NR) C: 2 (NR)	Ankylosing spondylitis OHIP‐14	E: Severe periodontitis: 46.0% (Page and Eke, 2007)^35^ C: No/mild periodontitis	Unadjusted	NR
Schmalz et al. 2018 ^18^ Germany Cross‐sectional	T: 60 (54.0, 50.0%) E: 22 (NR) C: 1 (NR)	Lung transplantation OHIP‐14	E: Severe periodontitis: 36.7% (Page and Eke, 2007)^35^ C: No/mild periodontitis	Unadjusted	NR
Schmalz et al. 2018 ^19^ Germany Cross‐sectional	Liver failure T: 24 (54.4, 41.7%) E: 8 (NR) C: 2 (NR) Liver transplantation T: 47 (56.6, 42.6%) E: 8 (NR) C: 12 (NR)	End‐stage liver failure and liver transplantation OHIP‐14	E: Severe periodontitis: 33.3% and 17.0% (Page and Eke, 2007)^35^ C: No/mild periodontitis	Unadjusted	NR
Mühlberg et al. 2017 ^16^ Germany Cross‐sectional	T: 103 (55.0, 56.3%) E: 13 (NR) C: 36 (NR)	Rheumatoid arthritis OHIP‐14	E: Severe periodontitis: 12.6% (Page and Eke, 2007)^35^ C: No/mild periodontitis	Unadjusted	NR
Schmalz et al. 2016 ^34^ Germany Cross‐sectional	Hemodialysis T: 87 (61.0, 37.9%) E: 84 (NR) C: 3 (NR) Kidney transplantation T: 39 (56.5, 51.3%) E: 34 (NR) C: 5 (NR)	End‐stage renal disease (hemodialysis) and Kidney transplantation OHIP‐14	E: Moderate/severe periodontitis: 96.6% and 87.2% (Page and Eke, 2007)^35^ C: No/mild periodontitis	Unadjusted	NR
Drumond‐Santana et al. 2007 ^11^ Brazil Cross‐sectional	T: 159 (NR, 65.4%) E: 34 (NR) C: 25 (NR)	Diabetes mellitus OHIP‐14	E: Severe periodontitis: 21.4% (PPD and CAL > 6 mm) C: No periodontitis	^**^Unadjusted	NR

Abbreviations: AAP, American Academy of Periodontology; CAL, clinical attachment level; CPI, community periodontal index; EFP, European Federation of Periodontology; NR, not reported; OHIP‐14, oral health impact profile; OHRQoL, oral health–related quality of life; OIDP, oral impact on daily performances; PPD, periodontal probing depth.

* Periodontitis definitions as reported in the primary studies.

** *p* < 0.05: statistically significant association.

### 3.2 Quantitative analysis

**FIGURE 2:**
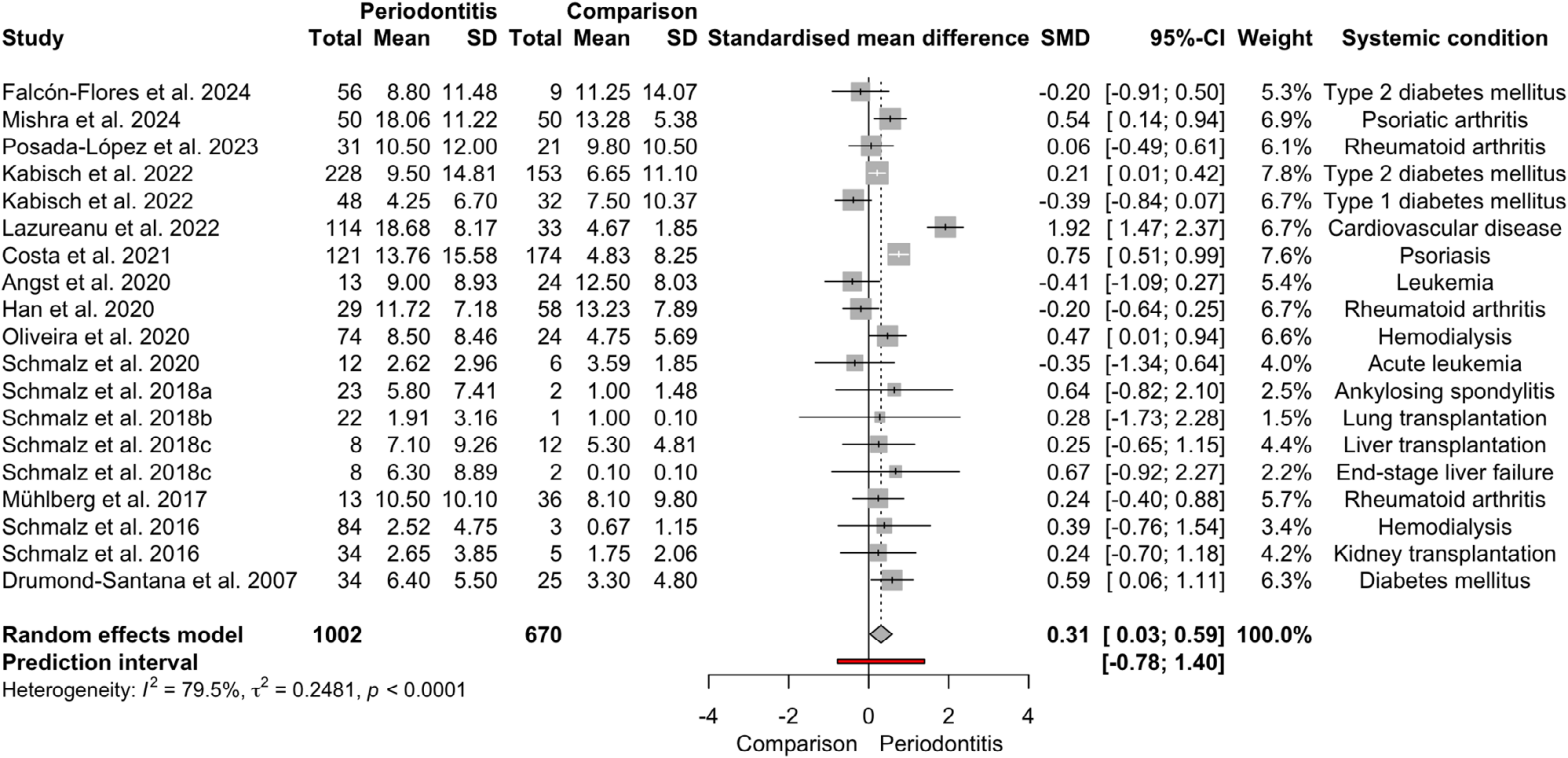
Forest plot of 16 studies (including three with data on different systemic diseases) that evaluated the association between periodontitis and OHRQoL in individuals with systemic medical conditions (SMD, standardized mean differences; CI, confidence intervals). *I^2^* (heterogeneity index) = 80% indicates significant heterogeneity, and a random‐effects model was applied.

**FIGURE 3:**
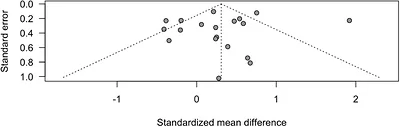
Funnel plot assessing publication bias of included studies.

Periodontitis impairs OHRQoL in patients with systemic conditions, however, the strength of this association is small (Figure 2; SMD: 0.31; 95% CI: 0.03–0.59; *p* = 0.029). The certainty level for this finding was deemed to be very low (see Table S4 in online *Journal of Periodontology*). Considerable heterogeneity was found among the studies (*I^2^* = 80%; *p* < 0.01). Similar results were found in the meta‐analysis of studies that used the OHIP‐14 instrument (see Figure S1 in online *Journal of Periodontology*; MD: 2.29; 95% CI: 0.24–4.33; *p* = 0.028; *I^2^*: 93%; *p* < 0.01). Despite the availability of adjusted regression analyses for continuous OHRQoL outcomes in some studies (Table 1), the meta‐analyses shown in Figures 2 and S1 were conducted using unadjusted mean and standard deviation data. The funnel plot is shown in Figure 3 and publication bias was not statistically significant (*p* = 0.558).

The meta‐regression analysis showed that comorbidity type and the periodontitis classification system partially explained the between‐study variability (*R^2^*: 54.1%; *p* = 0.013). Likewise, the same results were observed in the subgroup analyses in Table 2, regarding the type of comorbidity (*p* < 0.001) and the diagnosis of periodontitis (*p* = 0.034). Meta‐analysis of patients with psoriasis shows a strong negative impact of periodontitis on OHRQoL (SMD: 0.70; 95% CI: 0.39–1.01) (*p* < 0.001). Meta‐analyses of studies performed in patients with leukemia, rheumatoid arthritis or ankylosing spondylitis, end‐stage renal or liver diseases, diabetes or cardiovascular disease, and transplant recipients showed no association between periodontitis and OHRQoL (*p* > 0.05). Estimates from studies using the 2017 EFP/AAP periodontitis classification system were significantly higher (SMD: 0.83; 95% CI: 0.18–1.47) than those from studies applying earlier criteria (SMD: 0.10; 95% CI: −0.08–0.29) (*p* = 0.034). The inclusion of participants with periodontal attachment loss in comparison groups under older classification frameworks may have contributed to the lack of an observed association with OHRQoL.

**TABLE 2:** Pooled estimate of association between periodontitis and OHRQoL according to different subgroups (*n* = 16 studies and available data from 19 systemic conditions).

	*N*	SMD	95%CI	*p*	*I^2^* (%)	*p* ^***^ heterogeneity
**Overall** (All OHRQoL instruments)	19	0.31	0.03–0.59^*^	**<0.001** ^*^	80	**<0.01**
**Geographic location**				0.903		
Europe	11	0.38	−0.13–0.89		84	**<0.01**
Latin America	6	0.28	−0.09–0.65		71	**<0.01**
Asia	2	0.18	−0.54–0.90		83	**<0.01**
**Comorbidity**				**<0.001** ^**^		
Leukemia	2	−0.39	−1.24–0.46		0	0.92
Transplantation	3	0.25	−0.69–1.18		0	1.00
Rheumatoid arthritis or ankylosing spondylitis	4	0.01	−0.44–0.46		0	0.56
Diabetes or cardiovascular disease	5	0.43	−0.69–1.56		94	**<0.01**
End‐stage renal or liver diseases	3	0.47	−0.16–1.11		0	0.96
Psoriasis	2	0.70	0.39–1.01^*^		0	0.37
**Diagnosis of periodontitis**				**0.034** ^**^		
2017 EFP/AAP	4	0.83	0.18–1.47^*^		91	**<0.01**
Earlier criteria	15	0.10	−0.08–0.29		23	0.20
**Quality assessment**				0.371		
Moderate	10	0.19	−0.08–0.45		76	**<0.01**
Low	9	0.51	−0.15–1.18		77	**<0.01**

Abbreviations: CI, confidence interval; SMD, standardized mean difference; *I^2^*, heterogeneity index.

* *p* (combined estimate): *p* < 0.05 or a 95% confidence interval of the SMD that does not cross zero indicates a statistically significant association between periodontitis and OHRQoL.

** *p* (difference between subgroups): *p* < 0.05 indicates a statistically significant difference in effect estimates between subgroup categories.

^***^ *p* (heterogeneity): *p* < 0.05 indicates statistically significant heterogeneity.

**FIGURE 4:**
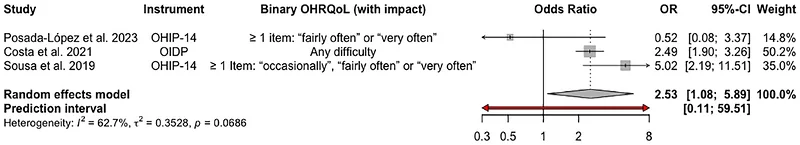
Forest plot of three studies (OR, adjusted model odds ratio; CI, confidence interval) showing the association between periodontitis and a negative impact on OHRQoL. *I^2^* (heterogeneity index) = 63% indicates moderate heterogeneity, and a random‐effects model was applied. The binary outcome was dichotomized according to the authors of each study (without or with impact on OHRQoL).

Binary OHRQoL data expressed as adjusted OR were available from three studies and were combined in a meta‐analysis (Figure 4). The studies considered different criteria to determine the impact on OHRQoL such as one or more items “fairly often” or “very often” to any question from the OHIP‐14,^33^ one or more items with the response “occasionally”, “fairly often”, or “very often” in the OHIP‐14^32^ or the occurrence of any difficulty in the OIDP instrument.^28^ Individuals with periodontitis were 2.53 times more likely to have a negative impact on OHRQoL compared with those without periodontitis (Figure 4. [log] OR = 2.53; 95% CI: 1.08 to 5.89; *p* = 0.032; *I^2^*:63%; *p* = 0.07) (certainty of evidence very low) (see Table S4 in online *Journal of Periodontology*). One study combined healthy individuals and patients with psoriasis when examining this association, which may have led to an overestimation of the results.^28^

## 4 DISCUSSION

This systematic review with meta‐analyses found that periodontitis negatively impacts OHRQoL in patients with systemic conditions. However, the pooled effect size was small. Differences in comorbidity type and in the diagnostic criteria used for periodontitis partially explained the substantial heterogeneity observed across studies. This is the first study to summarize information on this association in patients with systemic conditions.

Periodontitis symptoms such as swollen, painful, and receding gums, drifting and mobile teeth, and halitosis affect the physical, social, and psychological aspects of OHRQoL, negatively impacting function, comfort, appearance, and self‐confidence.^8^ Systematic reviews have shown a strong negative impact of periodontitis on OHRQoL in adults.^22^, ^39^, ^40^, ^41^ However, in this review, the results are somewhat different due to the small effect size, indicating that individuals with comorbidities appear to be more tolerant of periodontitis than the systemically healthy samples from previous reviews. A recent review reported a similar finding, though it did not assess which specific systemic conditions influence this association.^22^

The strong negative impact of periodontitis on OHRQoL observed in patients with psoriasis is likely explained by disease‐specific mechanisms and by the higher occurrence of periodontitis in this population. Psoriasis is a chronic systemic inflammatory condition that shares key immunoinflammatory pathways with periodontitis, including the overexpression of pro‐inflammatory cytokines such as interleukins and tumor necrosis factor‐α (TNF‐α), which contribute to sustained tissue destruction and chronic inflammation.^42^, ^43^ Immunological and genetic evidence indicates that both diseases involve dysregulated cytokine‐mediated adaptive immune responses, leading to self‐perpetuating inflammatory processes and overlapping etiopathogenic pathways.^44^, ^45^ Consistent with this interpretation, periodontitis appears to be more prevalent in patients with severe psoriasis and psoriatic arthritis.^44^, ^46^ In the study by Mishra et al.,^13^ included in this review, patients with psoriatic arthritis and generalized periodontitis exhibited significantly greater impairment in OHRQoL compared with other systemic conditions. Taken together, these findings suggest a synergistic interaction between psoriasis and periodontitis, amplifying the negative impact on OHRQoL, particularly in domains related to physical pain, psychological disability, and functional limitation.^13^, ^47^

Pooled estimates from studies of patients with other systemic diseases or conditions showed no association between periodontitis and OHRQoL. In these individuals, the perception of impact caused by periodontitis may not be important due to their higher vulnerability to other health problems, with greater severity and more debilitating outcomes than oral diseases. Thus, perceptions measured by OHRQoL instruments may not be strongly influenced by periodontal conditions, as the impact of periodontitis might be masked or overshadowed by the more serious or physically and emotionally debilitating diseases, such as leukemia.^21^, ^36^

The meta‐analysis of studies using the 2017 periodontitis classification showed significantly higher estimates than those based on earlier criteria. The 2017 EFP/AAP classification^4^ is characterized by high sensitivity, whereby a larger proportion of individuals are classified as having periodontitis due to a lower CAL threshold (1–2 mm), compared with other criteria commonly used in the included studies, such as the 2012 CDC/AAP criteria (CAL ≥ 3 mm combined with PPD measurements)^38^ or the Page and Eke^35^ definition (CAL ≥ 4 mm).^48^, ^49^ This improved sensitivity ensures that individuals classified as non‐periodontitis cases under the 2017 EFP/AAP criteria constitute a more homogeneous control group that is truly disease‐free, thereby minimizing exposure misclassification and enabling a more accurate detection of the impact of periodontitis on OHRQoL when comparing outcome scores between exposed and nonexposed groups. These findings underscore the critical role of diagnostic case definitions of periodontitis in epidemiological studies, as differences in exposure classification may substantially attenuate observed associations.

All studies, except Costa et al.,^28^ used the OHIP‐14, making comparisons between different OHRQoL measures unfeasible. The Oral Health Impact Profile^50^ is the most used instrument, widely tested and validated in adults, including periodontal patients, as it allows the relationship of different oral conditions with OHRQoL.^8^, ^24^ All measures tend to focus on dimensions relating to the physical/functional, psychological, and social impacts of oral conditions on the patient's life.^8^ In this review, the physical and psychological domains were the most impacted by periodontitis. However, the questions are not directly related to the condition of the periodontium, which can lead to incorrect answers, especially when the questionnaire is self‐administered. Other instruments were created for people with periodontitis (OHIP‐CP), and the questions included in them concern the most common symptoms and their impact on the patient's well‐being.^51^ However, this condition‐specific tool has not yet been compared with other instruments.

This review also has some limitations. The results should be interpreted with caution due to the limited number of studies, being only case‐control and cross‐sectional designs, and being of predominantly low to moderate quality. In addition, the review shows high heterogeneity, mainly due to the large variation in assessing exposure, types of comorbidities, and comparison groups, in addition to sociodemographic and regional characteristics. Due to the small number of studies, other subgroups did not explain the heterogeneity. Furthermore, when analyzing the different subgroups, the association between periodontitis and OHRQoL was not significant. Another limitation is that only studies that diagnosed periodontitis based on clinical periodontal parameters were included. This decision may influence the results, but these are the diagnostic parameters recommended by the current classification of periodontitis.^4^

Our findings may assist researchers and clinicians to have a better understanding of the impact of periodontitis on OHRQoL, considering both the type of systemic condition and the severity of periodontitis. These results underscore the importance of implementing targeted interventions to improve periodontal health.

## 5 CONCLUSION

Periodontitis has a small negative impact on OHRQoL in patients with systemic conditions. However, a stronger negative impact was observed in studies using the 2017 periodontitis classification and among individuals with systemic conditions that are less physically and emotionally debilitating, such as psoriasis. These findings should be interpreted with caution and a larger sample size, high‐quality studies, and evaluation of other systemic diseases are needed to confirm these results in future research.

## AUTHOR CONTRIBUTIONS

**Rafael Vargas Bortolaso**: Conceptualization; methodology; writing—original draft; writing—review and editing. **Catiusse Crestani Del**: Conceptualization; methodology; validation; formal analysis; investigation; data curation; writing—original draft; writing—review and editing. **Leandro Machado Oliveira**: Writing—review and editing. **Patricia A. Miguez**: Writing—review and editing. **Fabricio Batistin Zanatta**: Conceptualization; methodology; validation; formal analysis; investigation; writing—original draft; writing—review and editing; supervision. **Raquel Pippi Antoniazzi**: Conceptualization; methodology; validation; formal analysis; investigation; data curation; writing—original draft; writing—review and editing; supervision; project administration.

## CONFLICT OF INTEREST STATEMENT

The authors declare no conflicts of interest.

## Supporting information



SUPPORTING INFORMATION

SUPPORTING INFORMATION

SUPPORTING INFORMATION

SUPPORTING INFORMATION

SUPPORTING INFORMATION

SUPPORTING INFORMATION

SUPPORTING INFORMATION

## Data Availability

Data that support the findings of this study are available from the corresponding author upon reasonable request.
