# Supplementary material for: Association between periodontitis and oral health–related quality of life in patients with systemic conditions: A meta‐analysis
Source: J Periodontol. 2026 Apr 18;97(9):1990–2002. doi: 10.1002/jper.70130 (PMC13568998; doi:10.1002/jper.70130)
Supplement: Supplementary file 1 [file JPER-97-1990-s001.docx]

**Supplementary Table S2.** Search strategies presented according to each database.

| Database | Search strategy | Hits |
| --- | --- | --- |
| Pubmed/  MEDLINE  (https://www.ncbi.nlm.nih.gov/pubmed) | (((((((((((((((((((((((((((periodontal diseases[MeSH Terms]) OR (adult periodontitis[MeSH Terms])) OR (aggressive periodontitis[MeSH Terms])) OR (chronic periodontitis[MeSH Terms])) OR (attachment loss, periodontal[MeSH Terms])) OR (gingival pocket[MeSH Terms])) OR (periodontal pocket[MeSH Terms])) OR (periodontal attachment loss[MeSH Terms])) OR (diseases, periodontal)) OR (parodontosis)) OR (parodontoses)) OR (pyorrhea alveolaris)) OR (periodontitides)) OR (pericementitides)) OR (chronic periodontitides)) OR (adult periodontitides)) OR (periodontitis, prepubertal)) ) OR (prepubertal periodontitis)) OR (circumpubertal periodontitis)) OR (early-onset periodontitis)) OR (early onset periodontitis)) OR (juvenile periodontitis)) OR (juvenile periodontitides)) OR (periodontosis)) OR (periodontoses)) AND (((((((((((((((((((((((((((((((((((((((((((((((((((((((cardiovascular diseases[MeSH Terms]) OR (cardiovascular)) OR (diseases)) OR (cardiovascular disease)) OR (ischaemic heart disease)) OR (myocardial ischemia[MeSH Terms])) OR (myocardial)) OR (ischemia)) OR (ischemic)) OR (heart)) OR (disease)) OR (ischemic heart disease)) OR (coronary artery disease[MeSH Terms])) OR (coronary)) OR (artery)) OR (coronary artery disease)) OR (stroke)) OR (hypertensive)) OR (heart diseases)) OR (neoplasms)) OR (cancer)) OR (neoplasia)) OR (pulmonary disease, chronic obstructive)) OR (pulmonary)) OR (chronic obstructive pulmonary disease)) OR (copd[MeSH Terms]))) OR (respiratory tract infections)) OR (respiratory tract infections)) OR (respiratory infections)) OR (respiration disorders)) OR (diabetes mellitus[MeSH Terms])) OR (diabetes)) OR (mellitus))) OR (liver cirrhosis[MeSH Terms])) OR (cirrhosis)) OR (fibrosis)) OR (osteoarthritis[MeSH Terms])) OR (alzheimer disease[MeSH Terms])) OR (dementia)) OR (polypharmacy[MeSH Terms])) OR (multi morbidity)) OR (psoriatic arthritis[MeSH Terms])) OR (arthritis, psoriatic[MeSH Terms])) OR (psoriatic)) OR (rheumatoid arthritis[MeSH Terms])) OR (rheumatoid arthritis, splenomegaly and neutropenia[MeSH Terms])) OR (acute basophilic leukemia[MeSH Terms])) OR (leukemia)) OR (kidney disease)) OR (ankylosing spondylitis)) OR (transplantation)) OR (hemodialysis[MeSH Terms])) OR (dialysis[MeSH Terms]))) AND (((((((quality of life[MeSH Terms]) OR (life quality)) OR (health-related quality of life)) OR (health related quality of life)) OR (HRQOL)) OR (life qualities)) OR (oral health-related quality of life)) | 1810 |
| Embase  (https://www.embase.com) | ('`periodontal disease`' OR '`periodontal attachment loss`' OR '`periodontal near/4 disease`' OR 'periodontal near/4 diseases' OR '`periodontal near/4 infection`' OR '`periodontal tissue disease`' OR `periodontitis` OR '`chronic periodontitis`' OR '`aggressive periodontitis`' OR '`periodontal near/4 pocket`') AND ('cardiovascular disease'/exp OR 'ischemic heart disease'/exp OR 'ischemia'/exp OR 'coronary artery disease'/exp OR 'hypertension'/exp OR 'neoplasm'/exp OR 'malignant neoplasm'/exp OR cancer OR neoplasia OR 'chronic obstructive lung disease'/exp OR 'respiratory tract infection'/exp OR 'diabetes mellitus'/exp OR 'liver cirrhosis'/exp OR 'fibrosis'/exp OR 'osteoarthritis'/exp OR 'alzheimer disease' OR dementia OR polypharmacy OR 'multi morbidity' OR 'psoriasis'/exp OR 'psoriatic arthritis'/exp OR 'rheumatoid arthritis'/exp OR 'leukemia'/exp OR 'kidney disease'/exp OR 'ankylosing spondylitis'/exp OR 'transplantation'/exp OR hemodialysis OR dialysis) AND ('quality of life'/exp OR 'quality of life assessment'/exp OR 'life quality' OR 'health-related quality of life' OR 'life qualities' OR 'oral health related quality of life'/exp) | 846 |
| Scopus  (https://www.scopus.com) | ( ABS ( "periodontal diseases" OR "adult periodontitis" OR "aggressive periodontitis" OR "chronic periodontitis" OR "attachment loss, periodontal" OR "gingival pocket" OR "periodontal pocket" OR "periodontal attachment loss" OR "diseases, periodontal" OR "parodontosis" OR "parodontoses" OR "pyorrhea alveolaris" OR "periodontitides" OR "pericementitides" OR "chronic periodontitides" OR "adult periodontitides" OR "periodontitis, prepubertal" OR "prepubertal periodontitis" OR "circumpubertal periodontitis" OR "early-onset periodontitis" OR "early onset periodontitis" OR "juvenile periodontitis" OR "juvenile periodontitides" OR "periodontosis" OR "periodontoses" ) AND ABS ( "cardiovascular diseases" OR "cardiovascular" OR "diseases" OR "cardiovascular disease" OR "ischaemic heart disease" OR "myocardial ischemia" OR "myocardial" OR "ischemia" OR "ischemic" OR "heart" OR "disease" OR "ischemic heart disease" OR "coronary artery disease" OR "coronary" OR "artery" OR "coronary artery disease" OR "stroke" OR "hypertensive" OR "heart diseases" OR "neoplasms" OR "cancer" OR "neoplasia" OR "pulmonary disease, chronic obstructive" OR "pulmonary" OR "chronic obstructive pulmonary disease" OR "copd" OR "respiratory tract infections" OR "respiratory tract infections" OR "respiratory infections" OR "respiration disorders" OR "diabetes mellitus" OR "diabetes" OR "mellitus" OR "liver cirrhosis" OR "cirrhosis" OR "fibrosis" OR "osteoarthritis" OR "alzheimer disease" OR "dementia" OR "polypharmacy" OR "multi morbidity" OR "psoriatic arthritis" OR "arthritis, psoriatic" OR "psoriatic" OR "rheumatoid arthritis" OR "rheumatoid arthritis, splenomegaly and neutropenia" OR "acute basophilic leukemia" OR "leukemia" OR "kidney disease" OR "ankylosing spondylitis" OR "transplantation" OR "hemodialysis" OR "dialysis" ) AND ABS ( "quality of life" OR "life quality" OR "health-related quality of life" OR "health related quality of life" OR "HRQOL" OR "life qualities" OR "oral health-related quality of life" ) ) AND ( LIMIT-TO ( SUBJAREA , "DENT" ) ) | 333 |
| Web of Science  (https://login.webofknowledge.com) | abstract= (periodontal diseases OR adult periodontitis OR aggressive periodontitis OR chronic periodontitis OR attachment loss, periodontal OR gingival pocket OR periodontal pocket OR periodontal attachment loss OR diseases, periodontal OR parodontosis OR parodontoses OR pyorrhea alveolaris OR periodontitides OR pericementitides OR chronic periodontitides OR adult periodontitides OR periodontitis, prepubertal OR prepubertal periodontitis OR circumpubertal periodontitis OR early-onset periodontitis OR early onset periodontitis OR juvenile periodontitis OR juvenile periodontitides OR periodontosis OR periodontoses) AND abstract = (cardiovascular diseases OR cardiovascular OR diseases OR cardiovascular disease OR ischaemic heart disease OR myocardial ischemia OR myocardial OR ischemia OR ischemic OR heart OR disease OR ischemic heart disease OR coronary artery disease OR coronary OR artery OR coronary artery disease OR stroke OR hypertensive OR heart diseases OR neoplasms OR cancer OR neoplasia OR pulmonary disease, chronic obstructive OR pulmonary OR chronic obstructive pulmonary disease OR copd OR respiratory tract infections OR respiratory tract infections OR respiratory infections OR respiration disorders OR diabetes mellitus OR diabetes OR mellitus OR liver cirrhosis OR cirrhosis OR fibrosis OR osteoarthritis OR alzheimer disease OR dementia OR polypharmacy OR multi morbidity OR psoriatic arthritis OR arthritis, psoriatic OR psoriatic OR rheumatoid arthritis OR rheumatoid arthritis, splenomegaly and neutropenia OR acute basophilic leukemia OR leukemia OR kidney disease OR ankylosing spondylitis OR transplantation OR hemodialysis OR dialysis) AND abstract= (quality of life OR life quality OR health-related quality of life OR health related quality of life OR HRQOL OR life qualities OR oral health-related quality of life) | 814 |
| Lilacs  (lilacs.bvsalud.org) | (periodontal diseases OR adult periodontitis OR aggressive periodontitis OR chronic periodontitis OR attachment loss, periodontal OR gingival pocket OR periodontal pocket OR periodontal attachment loss OR diseases, periodontal OR parodontosis OR parodontoses OR pyorrhea alveolaris OR periodontitides OR pericementitides OR chronic periodontitides OR adult periodontitides OR periodontitis, prepubertal OR prepubertal periodontitis OR circumpubertal periodontitis OR early-onset periodontitis OR early onset periodontitis OR juvenile periodontitis OR juvenile periodontitides OR periodontosis OR periodontoses) AND (quality of life OR life quality OR health-related quality of life OR health related quality of life OR HRQOL OR life qualities OR oral health-related quality of life) | 20 |
| DANS  (https://easy.dans.knaw.nl/ui/datasets/id/easy-dataset:200362/tab/2) | Periodontal Diseases OR Chronic Periodontitis OR Aggressive Periodontitis OR Dental Caries OR Tooth Loss OR Xerostomia OR Oral Health OR Mouth Diseases and quality of life OR Life Quality OR Health-Related Quality Of Life OR Health Related Quality Of Life OR HRQOL OR Life Qualities | 217 |
| Total |  | 4.040 |
