# Supplementary material for: Association between periodontitis and oral health–related quality of life in patients with systemic conditions: A meta‐analysis
Source: J Periodontol. 2026 Apr 18;97(9):1990–2002. doi: 10.1002/jper.70130 (PMC13568998; doi:10.1002/jper.70130)
Supplement: Supplementary file 2 [file JPER-97-1990-s002.docx]

**Supplementary Figure S1.** Forest plot of fifteen studies (including three with data on different systemic diseases) that evaluated the association between periodontitis and OHRQoL in individuals with systemic medical conditions using the OHIP-14 instrument.

MD: mean difference; CI: confidence interval, I^2^: heterogeneity index.
