# Supplementary material for: Association between periodontitis and oral health–related quality of life in patients with systemic conditions: A meta‐analysis
Source: J Periodontol. 2026 Apr 18;97(9):1990–2002. doi: 10.1002/jper.70130 (PMC13568998; doi:10.1002/jper.70130)
Supplement: Supplementary file 3 [file JPER-97-1990-s003.docx]

**Supplementary Table S4.** Certainty of evidence assessed through GRADE approach (GRADEpro)

| **Certainty assessment** | | | | | | | **№ of patients** | | **Effect**  **(95% CI)** | **Certainty** |
| --- | --- | --- | --- | --- | --- | --- | --- | --- | --- | --- |
| **№ of studies** | **Study design** | **Quality**  **(NOS)** | **Inconsistency** | **Indirectness** | **Imprecision** | **Other considerations** | **Periodontitis** | **Comparison** |  |  |
| **Oral health–related quality of life (SMD: periodontitis *vs.* comparison). Figure 2 of the manuscript.** | | | | | | | | | | |
| 16 | Observational studies | Serious^a^ | Not serious | Not serious | Serious^b^ | - | 1002 | 670 | SMD: 0.31 CI: 0.03 -0.59 | ⨁◯◯◯ Very low |
| **Oral health–related quality of life (Adjusted OR):** Figure 3 of the manuscript. | | | | | | | | | | |
| 3 | Observational studies | Not serious | Serious^c^ | Not serious | Serious^b^ | - | 268 | 237 | OR: 2.53 CI: 1.08 -5.89 | ⨁◯◯◯ Very low |
| **Oral health–related quality of life (OHIP-14; Scale from: 0 to 56) (MD: periodontitis *vs.* comparison):** Supplementary Figure S7. | | | | | | | | | | |
| 15 | Observational studies | Very Serious^d^ | Serious^c^ | Not serious | Not Serious | - | 881 | 496 | MD: 2.29 CI: 0.24 -4.33 | ⨁◯◯◯ Very low |

CI: confidence interval; MD: mean difference; OR: Odds ratio; SMD: standardized mean difference

#### Explanations:

a. Between 25-50% of the weight of meta-analysis was composed by studies considered to have low quality.
b. The effect size is considered small (SMD< 0.5 and OR<3.47) (Chen et al., 2010; Schünemann et al. 2023).
c. Considerable heterogeneity was found among the studies and could not be explained (Adjusted OR: I^2^ = 63% and MD: I^2^=93%).
d. Between 25-50% of the weight of meta-analysis was composed by studies considered to have low quality.

**References:**

1. Chen H, Cohen P, Chen S. How big is a big odds ratio? Interpreting the magnitudes of odds ratios in epidemiological studies. Commun Stat Simul Comput. 2010;39:4,860-864. [doi.org/10.1080/03610911003650383](mailto:doi.org/10.1080/03610911003650383)
2. GRADEpro GDT: GRADEpro Guideline Development Tool [Software]. McMaster University and Evidence Prime, 2022. Available from [gradepro.org](https://gradepro.org/)
3. Schünemann HJ, Vist GE, Higgins JPT, Santesso N, Deeks JJ, Glasziou P, Akl EA, Guyatt GH. Chapter 15: Interpreting results and drawing conclusions [last updated August 2023]. In: Higgins JPT, Thomas J, Chandler J, Cumpston M, Li T, Page MJ, Welch VA (editors). Cochrane Handbook for Systematic Reviews of Interventions version 6.5. Cochrane, 2024. Available from [www.training.cochrane.org/handbook](http://www.training.cochrane.org/handbook)
