# Supplementary material for: Association between periodontitis and oral health–related quality of life in patients with systemic conditions: A meta‐analysis
Source: J Periodontol. 2026 Apr 18;97(9):1990–2002. doi: 10.1002/jper.70130 (PMC13568998; doi:10.1002/jper.70130)
Supplement: Supplementary file 4 [file JPER-97-1990-s004.docx]

**Supplementary Table S3.** Newcastle–Ottawa Scale (NOS)

**Newcastle-Ottawa quality assessment scale adapted for cross-sectional studies**

Note: A maximum of one star (*) is awarded for each numbered item and a maximum of two stars (**) are awarded for the items “Representativeness” and “Adjustment for confounding factors”.

**SELECTION (max. 2*)**

**1) Representativeness of the study sample:**

a) Truly representative of the average in the target population (all subjects or random sampling). **

b) Somewhat representative of the average in the target population (non-random sampling). *

c) Selected group of users.

d) No description of the sampling strategy.

**COMPARABILITY (max. 1*)**

**1) Comparison group:**

a) Comparison group including only individuals without periodontitis*

b) Comparison group composed of individuals with periodontal health, gingivitis and stage I-II periodontitis or equivalent in the same group.

**EXPOSURE (max. 2*)**

**1) Calibration for exposure (periodontitis):**

a) Calibrated examiner (description of training/calibration procedure) *

b) Non-calibrated examiner (absence of calibration or report of calibration, but with no description of procedure).

2) **Assessment of periodontitis:**

a) Adequate (diagnosis based on full mouth probing measurements, i.e., PPD and/or CAL) *

b) Inadequate (partial mouth recording or use of indexes with questionable value in describing the true periodontal status such as CPI)

d) Unclear (methods were not clear or not reported)

**OUTCOME (max. 4*)**

**1) Evaluation of outcome (oral health-related quality of life - OHRQoL):**

a) Blind evaluation (Assessment of quality of life with a self-administered instrument, administered by other researcher or before the clinical periodontal examination) *

b) Not blinded

**2) Adjustments for confounding factors:**

a) Association between periodontitis and OHRQoL adjusted for clinical confounding factors (xerostomia, dental caries, or tooth loss) **

b) Association between periodontitis and OHRQoL adjusted for other confounding factors *

c) None

**3) Response rate:**

a) All subjects selected for the study consented to be enrolled *****

b) Subjects lost are unlikely to introduce bias - small number lost (response rate ≥ 70%), or description provided of those lost) *****

c) Response rate < 70 % and no description of those lost.

d) No statement
