# Supplementary material for: Association between periodontitis and oral health–related quality of life in patients with systemic conditions: A meta‐analysis
Source: J Periodontol. 2026 Apr 18;97(9):1990–2002. doi: 10.1002/jper.70130 (PMC13568998; doi:10.1002/jper.70130)
Supplement: Supplementary file 6 [file JPER-97-1990-s006.docx]

**Supplementary Table S5.** List of studies excluded after evaluation of the full text, reasons for exclusion and studies without access to the full text.

| **Reason for Exclusion** | **Studies** |
| --- | --- |
| Participants: children or teenagers (*n* = 4) | [4, 12, 66, 74] |
| Periodontitis: self-reported, defined by radiographic parameters or unclear definition (*n* = 40) | [1, 2, 3, 6, 8, 10, 14,19, 26, 27, 32, 33, 34, 35, 36, 40, 42, 45, 47, 49, 54, 55, 56, 57, 58, 59, 63, 64, 65, 69, 72, 73, 75, 78, 79, 83, 84, 86, 87, 88] |
| OHRQoL: no validated instrument (*n* = 2) | [82, 85] |
| Study design: intervention study (*n* = 1) | [38] |
| Did not associate periodontitis with OHRQoL (*n* = 35) | [5, 7, 11, 13, 16, 17, 18, 20, 21, 24, 28, 30, 31, 37, 39, 41, 43, 44, 46, 48, 50, 51, 52, 53, 60, 61, 62, 67, 68, 71, 77, 80, 81, 89, 90] |
| No data for continuous or adjusted binary meta-analysis of patients with systemic diseases (n = 8) | [9, 15, 22, 23, 25, 29, 70, 76] |
| **Study references without access to the full text** | |
| [91, 92] | |

**REFERENCES (EXCLUDED STUDIES)**

1. Acharya S, Pentapati KC. Work stress and oral health-related quality of life among Indian information technology workers: an exploratory study. *Int Dent J.* 2012;62(3):132-136. doi:10.1111/j.1875-595X.2011.00104.x
2. Aloyouny AY, Almufarji F, Almutairi GG, Alkait S, Al-Mohaya MA, Alserwi R. Impact of rheumatic diseases on oral health-related quality of life. *Cureus.* 2022;14(12):e32268. doi:10.7759/cureus.32268
3. Amalia Z, Anggraeni ZK, Adiatman M. Association between oral health status and oral health-related quality of life in diabetes patients. *J Int Dent Med Res.* 2018;11:181-186.
4. Amaral Loureiro AC, Costa FO, Costa JE. The impact of periodontal disease on the quality of life of individuals with Down syndrome. *Down Syndr Res Pract.* 2007;12:50-54. doi:10.3104/reports.1998
5. Ancuta C, Antohe M, Ancuta E, Chirieac R, Iordache C. Oral health in patients with systemic sclerosis: an EUSTAR center experience. *Ann Rheum Dis.* 2017;76:1269. doi:10.1136/annrheumdis-2017-eular.4115
6. Ayan G, Dayi B. Evaluation of plaque index, gingival index and oral health-related quality of life in obese patients*. Odovtos Int J Dent Sci.* 2023;25(1):166-178. doi:10.15517/ijds.2022.52533
7. Baron M, Hudson M, Tatibouet S, Steele R, Lo E, Gravel S, Gyger G, El Sayegh T, Pope J, Fontaine A, Masseto A, Matthews D, Sutton E, Thie N, Jones N, Copete M, Kolbinson D, Markland J, Nogueira-Filho G, Robinson D, Gornitsky M. The Canadian systemic sclerosis oral health study: orofacial manifestations and oral health-related quality of life in systemic sclerosis compared with the general population. *Rheumatology (Oxford).* 2014;53:1386-1394. doi:10.1093/rheumatology/ket441
8. Bertl K, Tsakos G, Pandis N, Bogren A, Burisch J, Stavropoulos A. Health-related quality of life aspects of the 'Periodontitis prevalence in ulcerative colitis and Crohn's disease' (PPCC) cohort. *J Clin Periodontol.* 2023;50(12):1601-1620. doi:10.1111/jcpe.13863
9. Bertl K, Savvidis P, Kukla EB, Schneider S, Zauza K, Bruckmann C, Stavropoulos A. Including dental professionals in the multidisciplinary treatment team of head and neck cancer patients improves long-term oral health status. *Clin Oral Investig.* 2022;26(3):2937-2948. doi:10.1007/s00784-021-04276-x
10. Birungi N, Fadnes LT, Engebretsen IMS, Tumwine JK, Åstrøm AN, ANRS 12174 and 12341 Study Groups. The prevalence and socio-behavioural and clinical covariates of oral health-related quality of life in Ugandan mothers with and without HIV-1. *Health Qual Life Outcomes.* 2021;19:201. doi:10.1186/s12955-021-01844-3
11. Brasil-Oliveira R, Cruz ÁA, Souza-Machado A, Pinheiro GP, Inácio DDS, Sarmento VA, Lins-Kusterer L. Oral health-related quality of life in individuals with severe asthma. *J Bras Pneumol.* 2020;47:e20200117. doi:10.36416/1806-3756/e20200117
12. Busato IM, Ignácio SA, Brancher JA, Moysés ST, Azevedo-Alanis LR. Impact of clinical status and salivary conditions on xerostomia and oral health-related quality of life of adolescents with type 1 diabetes mellitus. *Community Dent Oral Epidemiol.* 2011;40:62-69. doi:10.1111/j.1600-0528.2011.00635.x
13. Camacho-Alonso F, Cánovas-García C, Martínez-Ortiz C, De la Mano-Espinosa T, Ortuño-Celdrán T, Marcello-Godino JI, Ramos-Sánchez R, Sánchez-Siles M. Oral status, quality of life, and anxiety and depression in hemodialysis patients and the effect of the duration of treatment by dialysis on these variables. *Odontology.* 2017;106:194-201. doi:10.1007/s10266-017-0313-6
14. Chen A, Ng ST, Goh V, Siu SC, Yeung K, Tsang YC, Wang Q, Leung WK. Assessing oral health and the minimally important differences in oral health-related quality of life of non-diabetic and diabetic patients: a cross-sectional study. *Aust Dent J.* 2024;69(3):206-218. doi:10.1111/adj.13017
15. Costa AA, Cota LOM, Esteves Lima RP, Oliveira AMSD, Cortelli SC, Cortelli JR, Cyrino RM, Mendes VS, Silva TA, Costa FO. The association between periodontitis and the impact of oral health on the quality of life of individuals with psoriasis and psoriatic arthritis. *PLoS One*. 2024;19(6):e0301158. doi:10.1371/journal.pone.0301158
16. Chiba FY, Sumida DH, Moimaz SAS, Chaves Neto AH, Nakamune ACMS, Garbin AJI, Garbin CAS. Periodontal condition, changes in salivary biochemical parameters, and oral health-related quality of life in patients with anorexia and bulimia nervosa. *J Periodontol.* 2019;90:1423-1430. doi:10.1002/JPER.19-0053
17. Ciardo A, Simon MM, Eberhardt R, Brock JM, Ritz A, Kim TS. Severe chronic obstructive pulmonary disease is associated with reduced oral health conditions. *Oral Dis.* 2023. doi:10.1111/odi.14755
18. Ciardo A, Simon MM, Awounvo S, Kim TS. Oral health conditions in patients under antiresorptive therapy are comparable to unexposed during supportive periodontal care. *Clin Oral Investig.* 2023;27(11):6523-6536. doi:10.1007/s00784-023-05257-y
19. Cicciù M, Matacena G, Signorino F, Brugaletta A, Cicciù A, Bramanti E. Relationship between oral health and its impact on the quality life of Alzheimer's disease patients: a supportive care trial. *Int J Clin Exp Med.* 2013;6:766-772.
20. Coates EA, Brennan D, Logan RM, Goss AN, Scopacasa B, Spencer AJ, Gorkic E. Hepatitis C infection and associated oral health problems. *Aust Dent J.* 2000;45:108-114. doi:10.1111/j.1834-7819.2000.tb00249.x
21. Coates E, Slade GD, Goss AN, Gorkic E. Oral conditions and their social impact among HIV dental patients. *Aust Dent J.* 1996;41:33-36. doi:10.1111/j.1834-7819.1996.tb05652.x
22. Cornejo C, Rossi G, Rama A, Gomez-Gutierrez N, Alvaredo G, Squassi A, Klemonskis G. Oral health status and oral health-related quality of life in pregnant women from socially deprived populations. *Acta Odontol Latinoam.* 2013;26(2):68-74.
23. Corrêa JD, Branco LGA, Calderaro DC, Mendonça SMS, Travassos DV, Ferreira GA, Teixeira AL, Abreu LG, Silva TA. Impact of systemic lupus erythematosus on oral health-related quality of life. *Lupus.* 2017;27(2):283-289. doi:10.1177/0961203317719147
24. de Azevedo Branco LG, Oliveira SR, Corrêa JD, Calderaro DC, Mendonça SMS, de Queiroz Cunha F, Ferreira GA, Teixeira AL, Abreu LG, Silva TA. Oral health-related quality of life among individuals with rheumatoid arthritis*. Clin Rheumatol.* 2019;38:2433-2441. doi:10.1007/s10067-019-04555-9
25. de Oliveira Araújo R, Villoria GEM, Luiz RR, Esteves JC, Leão ATT, Feres-Filho EJ. Association between periodontitis and Alzheimer's disease and its impact on the self-perceived oral health status: a case-control study. *Clin Oral Investig.* 2021;25(2):555-562. doi:10.1007/s00784-020-03489-w
26. de Pinho AM, Borges CM, de Abreu MH, Ferreira EF, Vargas AM. Impact of periodontal disease on the quality of life of diabetics based on different clinical diagnostic criteria. *Int J Dent.* 2012;2012:986412. doi:10.1155/2012/986412
27. Farran M, Neppelberg E, Løes S, Aarstad AKH, Moe SE, Aarstad HJ. Periodontitis and dental quality of life predict long-term survival in head and neck cancer. *BMC Oral Health.* 2024;24(1):1406. doi:10.1186/s12903-024-05170-0
28. Garbade J, Rast J, Schmalz G, Eisner M, Wagner J, Kottmann T, Oberbach A, Lehmann S, Haak R, Borger MA, Binner C, Ziebolz D. Oral health and dental behaviour of patients with left ventricular assist device: a cross-sectional study. *ESC Heart Fail.* 2020;3:1273-1281. doi:10.1002/ehf2.12636
29. Geevarghese A, Baskaradoss JK, Sarma PS. Oral health-related quality of life and periodontal status of pregnant women. *Matern Child Health J.* 2017;21(8):1634-1642. doi:10.1007/s10995-016-2255-y
30. Glavina A, Božić I, Parat K, Perković D, Biočina-Lukenda D, Martinović Kaliterna D, Radić M. The Croatian Primary Sjögren's Disease Oral Health Study: oral status and oral health-related quality of life. *J Clin Med.* 2023;12(14):4804. doi:10.3390/jcm12144804
31. Glavina A, Božić I, Parat K, Perković D, Biočina-Lukenda D, Martinović Kaliterna D, Radić M. Salivary flow rate and oral status in patients with primary Sjögren's syndrome and diffuse cutaneous systemic sclerosis: a cross-sectional study. *Diagnostics (Basel).* 2023;13(6):1057. doi:10.3390/diagnostics13061057
32. Guzeldemir E, Toygar HU, Tasdelen B, Torun D. Oral health-related quality of life and periodontal health status in patients undergoing hemodialysis. *J Am Dent Assoc.* 2009;140:1283-1293. doi:10.14219/jada.archive.2009.0052
33. Hajian-Tilaki A, Oliae F, Jenabian N, Hajian-Tilaki K, Motallebnejad M. Oral health-related quality of life and periodontal and dental health status in Iranian hemodialysis patients. *J Contemp Dent Pract.* 2014;15:482-490. doi:10.5005/jp-journals-10024-1566
34. Hammad RN, Ahmad SA, Rasool MI. Impact of biologic and disease-modifying anti-rheumatic drug (DMARD) therapies on oral health in rheumatologic patients: a case-control study. *Cureus.* 2024;16(11):e73179. doi:10.7759/cureus.73179
35. Hanisch M, Blanck-Lubarsch M, Bohner L, Suwelack D, Kleinheinz J, Köppe J. Oral conditions and oral health-related quality of life of people with Ehlers-Danlos syndromes (EDS): a questionnaire-based cross-sectional study. *Medicina (Kaunas).* 2020;56:448. doi:10.3390/medicina56090448
36. Haresaku S, Nakashima F, Hara Y, Kuroki M, Aoki H, Kubota K, Naito T. Associations of oral health-related quality of life with age, oral status, and oral function among psychiatric inpatients in Japan: a cross-sectional study. *BMC Oral Health.* 2020;20:361. doi:10.1186/s12903-020-01355-5
37. Haugbo HO, Klepp P, Verket A. Ulcerative colitis and periodontitis - a cross-sectional pilot study from a Norwegian cohort. *Acta Odontol Scand.* 2023;81(7):541-548. doi:10.1080/00016357.2023.2210660
38. Irani FC, Wassall RR, Preshaw PM. Impact of periodontal status on oral health-related quality of life in patients with and without type 2 diabetes. *J Dent.* 2015;43:506-511. doi:10.1016/j.jdent.2015.03.001
39. Jung YS, Park EY, Sohn HO. Oral health status and oral health-related quality of life according to presence or absence of mucositis in head and neck cancer patients. *J Cancer Prev.* 2019;24:43-47. doi:10.15430/JCP.2019.24.1.43
40. Katkam P, Puranik MP, Kr S. Association between oral health status and oral health-related quality of life in individuals with thyroid dysfunction: a cross-sectional study. *Cureus.* 2024;16(11):e74595. doi:10.7759/cureus.74595
41. Khongsirisombat N, Kiattavorncharoen S, Thanakun S. Increased oral dryness and negative oral health-related quality of life in older people with overweight or obesity. *Dent J.* 2022;10(12):231. doi:10.3390/dj10120231
42. Kroese JM, Volgenant CMC, van Schaardenburg D, van Boheemen L, van Selms MKA, Visscher CM, Crielaard W, Loos BG, Lobbezoo F. Oral health-related quality of life in patients with early rheumatoid arthritis is associated with periodontal inflammation and painful temporomandibular disorders: a cross-sectional study. *Clin Oral Investig.* 2021;26:555-563. doi:10.1007/s00784-021-04034-z
43. Kwon EY, Joo J. Assessment of oral health-related quality of life and periodontal state in patients with end-stage renal disease: a comparison study using propensity score-matched analysis. *Med Oral Patol Oral Cir Bucal.* 2024;29(6):e768-e774. doi:10.4317/medoral.26714
44. Lalla RV, Long-Simpson L, Hodges JS, Treister N, Sollecito T, Schmidt B, Patton LL, Brennan MT, OraRad Study Group. Clinical registry of dental outcomes in head and neck cancer patients (OraRad): rationale, methods, and recruitment considerations. *BMC Oral Health.* 2017;17:59. doi:10.1186/s12903-017-0344-y
45. Li Z, Zhu L, Sha Y. Effects of periodontal health and related factors on the oral health-related quality of life in type 2 diabetic patients with chronic periodontitis. *Hua Xi Kou Qiang Yi Xue Za Zhi.* 2011;29:379-383.
46. Liberali SA, Coates EA, Freeman AD, Logan RM, Jamieson L, Mejia G. Oral conditions and their social impact among HIV dental patients, 18 years on. *Aust Dent J.* 2013;58:18-25. doi:10.1111/adj.12031
47. Lu HX, Xu W, Wong MC, Wei TY, Feng XP. Impact of periodontal conditions on the quality of life of pregnant women: a cross-sectional study. *Health Qual Life Outcomes.* 2015;13:67. doi:10.1186/s12955-015-0267-8
48. Machado V, Botelho J, Proença L, Alves R, Oliveira MJ, Amaro L, Águas A, Mendes JJ. Periodontal status, perceived stress, diabetes mellitus and oral hygiene care on quality of life: a structural equation modelling analysis. *BMC Oral Health.* 2020;20:229. doi:10.1186/s12903-020-01219-y
49. Marquezin MCS, Chaves-Júnior SC, Rasera IJr, Pacheco ERP, Gavião MBD, Lamy E, Castelo PM. Oral health and nutritional characteristics of adults with morbid obesity: a multivariate analysis. *Front Nutr.* 2020;7:589510. doi:10.3389/fnut.2020.589510
50. Marsicano JA, Grec PG, Belarmino LB, Ceneviva R, Peres SH. Interfaces between bariatric surgery and oral health: a longitudinal survey. *Acta Cir Bras.* 2011;2:79-83. doi:10.1590/s0102-86502011000800015
51. Martínez-Beneyto Y, Montero-Martin J, Garcia-Navas F, Vicente-Hernandez A, Ortiz-Ruiz AJ, Camacho-Alonso F. Influence of a preventive program on the oral health-related quality of life (OHRQoL) of European pregnant women: a cohort study. *Odontology.* 2018;107:10-16. doi:10.1007/s10266-018-0356-3
52. Mohamed HG, Idris SB, Ahmed MF, Bøe OE, Mustafa K, Ibrahim SO, Astrøm AN. Association between oral health status and type 2 diabetes mellitus among Sudanese adults: a matched case-control study. *PLoS One.* 2013;8:e82158. doi:10.1371/journal.pone.0082158
53. Mohamed HG, Mustafa K, Ibrahim SO, Åstrøm AN. Dietary habits, oral impact on daily performance and type 2 diabetes: a matched case-control study from Sudan. *Health Qual Life Outcomes.* 2017;15:111. doi:10.1186/s12955-017-0686-9
54. Moimaz SA, Rocha NB, Garbin AJ, Garbin CA, Saliba O. Influence of oral health on quality of life in pregnant women. *Acta Odontol Latinoam*. 2016;29:186-193.
55. Molania T, Salehi M, Ehsani H, Moosazadeh M, Niksolat F, Rezaei A, Salehabadi N, Rezaei F, Ebrahimi Saravi M. Comparison of periodontal indices, DMFT, xerostomia, hyposalivation and oral health-related quality of life in Sjögren's syndrome patients versus healthy individuals: a case-control study. *Dent Med Probl.* 2023;60(1):99-107. doi:10.17219/dmp/146771
56. Molania T, Malekzadeh Shafaroudi A, Taghavi M, Ehsani H, Moosazadeh M, Haddadi A, Gholizadeh N, Salehi M. Oral health-related quality of life (OHRQoL) in cardiovascular patients referring to Fatima Zahra Hospital in Sari, Iran. *BMC Oral Health.* 2021;21:391. doi:10.1186/s12903-021-01756-0
57. Mulligan R, Seirawan H, Alves ME, Navazesh M, Phelan JA, Greenspan D, Greenspan JS, Mack WJ. Oral health-related quality of life among HIV-infected and at-risk women. *Community Dent Oral Epidemiol.* 2008;36:549-557. doi:10.1111/j.1600-0528.2008.00443.x
58. Mumcu G, Niazi S, Stewart J, Hagi-Pavli E, Gokani B, Seoudi N, Ergun T, Yavuz S, Stanford M, Fortune F, Direskeneli H. Oral health and related quality of life status in patients from UK and Turkey: A comparative study in Behcet's disease. *J Oral Pathol Med.* 2009;38:406-409. doi:10.1111/j.1600-0714.2009.00752.x
59. Murererehe J, Malele-Kolisa Y, Niragire F, Yengopal V. Oral health-related quality of life among people living with HIV and HIV-negative adults in Kigali, Rwanda: A comparative cross-sectional study. *BMC Oral Health.* 2024;24(1):128. doi:10.1186/s12903-023-03828-9
60. Oduncuoğlu BF, Alaaddinoğlu EE, Çolak T, Akdur A, Haberal M. Effects of renal transplantation and hemodialysis on patient's general health perception and oral health-related quality of life: A single-center cross-sectional study. *Transplant Proc.* 2020;52:785-792. doi:10.1016/j.transproceed.2020.01.016
61. Olofsson R, Korytowska M, Almhöjd U, Almståhl A, Cevik-Aras H. Oral health, dental treatment, and medication related osteonecrosis of the jaw in multiple myeloma - A longitudinal cohort study. *BMC Oral Health.* 2024;24(1):184. doi:10.1186/s12903-024-03943-1
62. Oluwatoyin AE, Arinola E, Olufemi OE, Jokotade A. Self-reported oral health and oral health-related quality of life among patients with diabetes mellitus in a tertiary health facility. *BMC Oral Health.* 2024;24(1):181. doi:10.1186/s12903-023-03336-w
63. Pakpour AH, Kumar S, Fridlund B, Zimmer S. A case-control study on oral health-related quality of life in kidney disease patients undergoing haemodialysis. *Clin Oral Investig.* 2014;19:1235-1243. doi:10.1007/s00784-014-1355-6
64. Panzarella V, Mauceri R, Baschi R, Maniscalco L, Campisi G, Monastero R. Oral health status in subjects with amnestic mild cognitive impairment and Alzheimer's disease: Data from the Zabút Aging Project. *J Alzheimers Dis.* 2022;87:173-183. doi:10.3233/JAD-200385
65. Patel R, Gamboa A. Prevalence of oral diseases and oral-health-related quality of life in people with severe mental illness undertaking community-based psychiatric care. *Br Dent J.* 2012;213:E16. doi:10.1038/sj.bdj.2012.989
66. Polizzi A, Santonocito S, Di Stefano M, Ferlito S, Indelicato F, Palazzo G. The effects on oral related quality of life induced by periodontitis in patients with juvenile idiopathic arthritis. *Mediterr J Clin Psychol.* 2020;8:1-16. doi:10.6092/2282-1619/mjcp-2426
67. Polizzi A, Santonocito S, Vaccaro M, Fichera G, Torrisi S, Ronsivalle V, Palazzo G, Sicari F, Indelicato F. Relationship between periodontitis and psychosocial impact in patients with systemic sclerosis: A clinical study. *Mediterr J Clin Psychol.* 2020;8:1-21. doi:10.6092/2282-1619/mjcp-2509
68. Radujković V, Lovrenčić-Huzjan A, Puhar I. Periodontal disease in patients with ischemic stroke - an exploratory study. *Acta Stomatol Croat.* 2024;58(2):110-122. doi:10.15644/asc58/2/1
69. Reyes Garita P, Tran VT, Chatzopoulou E, Toko-Kamga L, Bouchard P, Carra MC. Oral hygiene behaviors and periodontitis among patients with chronic diseases and its impact on tooth loss and oral health-related quality of life: A cross-sectional study of data from the ComPaRe e-cohort. *Clin Oral Investig.* 2024;28(10):518. doi:10.1007/s00784-024-05903-z
70. Roumeau S, Thevenon J, Ouchchane L, Maqdasy S, Batisse-Lignier M, Duale C, Pham Dang N, Caron P, Tauveron I, Devoize L. Assessment of oro-dental manifestations in a series of acromegalic patients, the AcroDent study. *Endocr Connect.* 2020;9(8):824-833. doi:10.1530/EC-20-0176
71. Sales-Peres SHC, Groppo FC, Bonato RCS, Sales-Peres MC, Haiter-Neto F, Chaim EA. Alveolar bone pattern and salivary leptin levels among premenopausal obese women. *Arq Bras Cir Dig.* 2019;32:e1422. doi:10.1590/0102-672020180001e1422
72. Samuel SR, Kuduruthullah S, Khair AMB, Al Shayeb M, Elkaseh A, Varma SR, Nadeem G, Elkhader IA, Ashekhi A. Impact of pain, psychological-distress, SARS-CoV2 fear on adults' OHRQoL during COVID-19 pandemic. *Saudi J Biol Sci.* 2020;28:492-494. doi:10.1016/j.sjbs.2020.10.033
73. Sánchez GA, D'Eramo LR, Lecumberri R, Squassi AF. Impact of oral health care needs on health-related quality of life in adult HIV+ patients. *Acta Odontol Latinoam.* 2011;24:92-97.
74. Santana NCM, Rocha PAD, de Arruda JAA, Pascoal TP, Prado HV, Costa ACM, Costa FO, Torres-Pereira CC, Abreu LG, Fournier BPJ, Silva TA. Oral health-related quality of life in Fanconi anemia: A cross-sectional study. *Support Care Cancer.* 2024;32(9):571. doi:10.1007/s00520-024-08777-9
75. Santo AE, Tagliaferro EP, Ambrosano GM, Meneghim MC, Pereira AC. Dental status of Portuguese HIV+ patients and related variables: A multivariate analysis. *Oral Dis.* 2009;16:176-184. doi:10.1111/j.1601-0825.2009.01622.x
76. Santos RC, Pinho RCM, Cimões R. Chronic periodontitis in patients with type 2 diabetes: Analysis of the FokI polymorphism and perception of quality of life. *Pesq Bras Odontopediatria Clin Integr*. 2020;20:e4395. doi:10.1590/pboci.2020.149
77. Santos PS, Cremonesi AL, Quispe RA, Rubira CM. The impact of oral health on quality of life in individuals with head and neck cancer after radiotherapy: The importance of dentistry in psychosocial issues. *Acta Odontol Latinoam.* 2017;30:62-67.
78. Schmalz G, Ziebolz H, Kottmann T, Ziebolz D, Laskawi R. Oral health and oral health-related quality of life in patients with oral dystonia indicates their need for dental special care. *Med Oral Patol Oral Cir Bucal.* 2021;26:e554-e560. doi:10.4317/medoral.24479.
79. Schmalz G, Binner C, Eisner M, Wagner J, Rast J, Kottmann T, Haak R, Lehmann S, Borger MA, Garbade J, Ziebolz D. Oral health-related quality of life in patients with heart failure and left ventricular assist devices-results of a cross-sectional study. *Clin Oral Investig.* 2021;25:5879-5887. doi:10.1007/s00784-021-03893-w.
80. Schmalz G, Denkler CR, Kottmann T, Rinke S, Ziebolz D. Oral Health-Related Quality of Life, Oral Conditions, and Risk of Malnutrition in Older German People in Need of Care-A Cross-Sectional Study. *J Clin Med.* 2021;10:426. doi:10.3390/jcm10030426.
81. Schmalz G, Noack S, Patschan S, Patschan D, Müller GA, Rupprecht A, Schmickler J, Haak R, Ziebolz D. Disease activity, morning stiffness and missing teeth are associated with oral health-related quality of life in individuals with rheumatoid arthritis. *Clin Oral Investig.* 2020;24:3559-3566. doi:10.1007/s00784-020-03226-3.
82. Singh A, Long SM, Tin AL, Vickers AJ, Yom SK, Lee NY, Huryn JM, Cracchiolo JR, Estilo CL. Association between objective measures of oral health and salivary gland irradiation with patient-reported outcomes following head and neck radiation therapy. *Head Neck.* 2024;46(12):3066-3075. doi:10.1002/hed.27884.
83. Tassanapong N, Valenzuela Torres O, Myat Thwin K, Ogawa H. Psychosocial Determinants of Oral Health Related to COVID-19 Infection in Myanmar Older Adults. *Asia Pac J Public Health.* 2024;37(1):69-77. doi:10.1177/1010539524129665.
84. Tristiu R, Vesa S, Dumitru RB, Arweiler NB, Cosgarea RM, Lascu L, Rednic S, Eick S, Sculean A, Cosgarea R. Association of Oral-Health Related Quality of Life and General Health Assessment in Patients with Rheumatoid Arthritis. *Oral Health Prev Dent.* 2018;16:271-280. doi:10.3290/j.ohpd.a39912.
85. Viñas P, Martín-Martínez A, Cera M, Riera SA, Escobar R, Clavé P, Ortega O. Characteristics and therapeutic needs of older patients with oropharyngeal dysphagia admitted to a general hospital. *J Nutr Health Aging.* 2023;27(11):996-1004. doi:10.1007/s12603-023-1996-8.
86. Vu GT, Little BB, Esterhay RJ, Jennings JA, Creel L, Gettleman L. Oral health-related quality of life in US adults with type 2 diabetes. *J Public Health Dent.* 2022;82:79-87. doi:10.1111/jphd.12489.
87. Wandera MN, Engebretsen IM, Rwenyonyi CM, Tumwine J, Astrøm AN, PROMISE-EBF Study Group. Periodontal status, tooth loss and self-reported periodontal problems effects on oral impacts on daily performances, OIDP, in pregnant women in Uganda: a cross-sectional study. *Health Qual Life Outcomes.* 2009;7:89. doi:10.1186/1477-7525-7-89.
88. Yakşi E, Horasan N. Vitamin D levels and oral health in stroke patients during inpatient rehabilitation. *J Oral Rehabil.* 2023;50(4):293-299. doi:10.1111/joor.13415.
89. Yamashita JM, Moura-Grec PG, Freitas AR, Sales-Peres A, Groppo FC, Ceneviva R, Sales-Peres SH. Assessment of Oral Conditions and Quality of Life in Morbid Obese and Normal Weight Individuals: A Cross-Sectional Study. *PLoS One.* 2015;10:e0129687. doi:10.1371/journal.pone.0129687.
90. Ye N, Deng B, Hu H, Ai Y, Liu X, Zhou S, Li Y. The association between oral health and mild cognitive impairment in community-dwelling older adults. *Front Public Health*. 2024;12:1464439. doi:10.3389/fpubh.2024.1464439.

**REFERENCES (STUDY REFERENCES WITHOUT ACCESS)**

91. Paçô MTP, Gomes MJ, Teixeira C. The impact of oral health on quality of life in type-2 diabetic older people from Inland Northern Portugal. *Aten Primaria*. 2014;46:54-54.
92. Suppiah P, Cullinan MP, Nolan A, Thompson WM, Stebbings S. An association between periodontal disease, oral health related quality of life and disease activity in patients with ankylosing spondylitis. *Rheumatology.* 2013;52.
