# Supplementary material for: Association between periodontitis and oral health–related quality of life in patients with systemic conditions: A meta‐analysis
Source: J Periodontol. 2026 Apr 18;97(9):1990–2002. doi: 10.1002/jper.70130 (PMC13568998; doi:10.1002/jper.70130)
Supplement: Supplementary file 7 [file JPER-97-1990-s007.docx]

**Supplementary Table S6.** Quality assessment of the included studies based on the number of stars (points) assigned according to the Newcastle–Ottawa Scale adapted for cross-sectional studies (n = 17).

| Cross-sectional Study | Selection | Comparability | Exposition | Outcome | Max 9 * | Max 100% | Quality |
| --- | --- | --- | --- | --- | --- | --- | --- |
| 1. Falcón-Flores et al. 2024 ^37^ | -- | * | ** | * | 4/9 | 44.4% | Moderate |
| 2. Mishra et al. 2024 ^13^ | -- | * | ** | * | 4/9 | 44.4% | Moderate |
| 3. Posada-López et al. 2023 ^33^ | -- | * | ** | ** | 5/9 | 55.5% | Moderate |
| 4. Kabisch et al. 2022 ^52^ | * | * | -- | *** | 5/9 | 55.5% | Moderate |
| 5. Lazureanu et al. 2022 ^14^ | -- | * | -- | ** | 3/9 | 33.3% | Low |
| 6. Costa et al. 2021 ^28^ | -- | * | ** | ** | 5/9 | 55.5% | Moderate |
| 7. Angst et al. 2020 ^36^ | -- | -- | ** | ** | 4/9 | 44.4% | Moderate |
| 8. Han et al. 2020 ^20^ | -- | * | ** | ** | 5/9 | 55.5% | Moderate |
| 9. Oliveira et al. 2020 ^12^ | * | * | ** | * | 5/9 | 55.5% | Moderate |
| 10. Schmalz et al. 2020 ^21^ | -- | -- | * | -- | 1/9 | 11.1% | Low |
| 11. Sousa et al. 2019 ^32^ | ** | * | ** | **** | 9/9 | 100.0% | High |
| 12. Schmalz et al. 2018a ^17^ | ***--*** | - | ***** | -- | 1/9 | 11.1% | Low |
| 13. Schmalz et al. 2018b ^18^ | -- | -- | * | -- | 1/9 | 11.1% | Low |
| 14. Schmalz et al. 2018c ^19^ | -- | -- | ******* | -- | 1/9 | 11.1% | Low |
| 15. Mühlberg et al. 2017 ^16^ | **--** | * | * | * | 3/9 | 33.3% | Low |
| 16. Schmalz et al. 2016 ^34^ | -- | -- | * | -- | 1/9 | 11.1% | Low |
| 17. Drumond-Santana et al. 2007 ^11^ | -- | * | ** | ** | 5/9 | 55.5% | Moderate |
